# High-efficiency, flexibility and lead-free X-ray shielding multilayered polymer composites: layered structure design and shielding mechanism

**DOI:** 10.1038/s41598-021-83031-4

**Published:** 2021-02-23

**Authors:** Zefeng Li, Wei Zhou, Xianlong Zhang, Yue Gao, Shaoyun Guo

**Affiliations:** 1 The State Key Laboratory of Polymer Materials Engineering, Polymer Research Institute of Sichuan University, Chengdu, 610065 China; 2 Department of Pharmaceutical Sciences, Beijing Institute of Radiation Medicine, Beijing, 100850 China

**Keywords:** Materials science, Other photonics

## Abstract

To overcome the severe toxicity and blind absorption zone of conventional lead-based shielding materials for X-rays in the 70–90 keV range, the lead-free multilayered polymer composites were designed and fabricated. The effects of the direction of incidence of the X-rays and number of layers as well as layer thickness ratio of the (tungsten/ethylene-octene copolymer)/(bismuth/ethylene-octene copolymer) layered composites on their shielding efficiency were studied systematically. Compared to the traditional polymer blending, the multilayered polymer composites exhibited the improved photon attenuation. The multilayered polymer composites (layer thickness ratio was 3:7) with 6 layers had the best X-ray shielding ability. Moreover, the X-ray shielding provided by the multi-layered interfaces and the multiple complementary effect of the absorption within the multilayered structure were firstly proposed based on computer simulations. The multilayered structural design effectively weakened the probability of the X-ray penetration. Therefore, the X-ray shielding capability can be effectively enhanced through increasing number of layers and the synergistic effect of multi-layered interfaces. The experimental results of this study can serve as guidelines for the fabrication of flexibility, lead-free, lightweight and high-efficiency X-ray shielding materials.

## Introduction

X-ray, which is strongly ionizing, is used widely for nondestructive testing, medical diagnosis, medical radiotherapy, and geological exploration, as well as in security systems. Because of the biological effects of X-rays, its hazards have always attracted heightened attention. In recent years, X-rays had been listed as being carcinogenic by the International Committee on Radiological Protection (ICRP)^1,2^. Therefore, shielding X-rays to weaken its carcinogenic effects as well as its genetic effects, which can cause cell mutations, has become an urgent issue in the field of radiation protection^3,4^.

X-ray photons are low-energy photons. Hence, X-ray shielding primarily depends on the probability of the photoelectric and Compton scattering effects occurring between the incident photons and the shielding material. For certain materials, the absorption coefficient increases sharply when the incident photons interact with the inner-shell electrons. The corresponding energy is called the absorption limit or absorption edge of the material in question. The eventual dissipation of the photon energy occurs entirely owing to the photoelectric effect. If the shielding material has a large number of electrons and multiple absorption edges, its ability to shield photons with different energies is significantly improved owing to the photoelectric effect^5,6^.

Lead is the most widely used shielding material, because its ability to efficiently shield X-rays is superior to that of other materials owing to its high Z number, high density, and low cost. However, lead-based shielding materials usually have a few significant drawbacks, such as high toxicity, heavy nature, poor flexibility, and low chemical stability. More importantly, they exhibit a blind absorption zone for X-rays in the 70 − 90 keV range^7^. Therefore, novel shielding materials are urgently required to replace conventional lead-containing ones.

In the past decades, there have several reports on the use of composites containing (tungsten) W and (Bismuth) Bi micro/nanoparticles to attenuate high-energy radiation. Given the arrangement of atoms with different absorption edges and the high specific surface area of nanoparticles, polymer composites filled with metallic nanoparticles exhibit greater shielding effectiveness than that of polymer composites filled with microparticles^8–12^. Noor Azman et al*.* used bismuth or bismuth oxide, tungsten, tungsten carbide, tungsten oxide, bismuth tungstate, nanogold, nanosilver, and graphene as replacements for lead and lead-based compounds and fabricated polymer composites for shielding X-rays^13–15^. Importantly, the absorption blindness of these lead-based shielding materials in 70–90 keV range was offset in the case of the polymer composites by the compounding effect of the functional fillers, which were of different types and whose particles were of different sizes.

In order to further enhance the shielding ability of polymer composites, Hu et al.^16^ used a genetic algorithm to design and fabricate a shielding material with three layered structure (iron-lead-iron sandwich structure), wherein the characteristics of the three layered structure were fully exploited to ensure that the material exhibited outstanding shielding ability with respect to γ-rays and neutrons. In addition, using computer simulations, Fan et al.^17^ showed that laminated sheet fillers exhibit excellent shielding ability with respect to high-energy photons. Based on their results, Kim^18^ prepared layered ethylene-octene copolymer (POE) composites with W and flakes fillers. These composites showed excellent shielding ability for X-rays. Further, using the group optimization algorithm, Sazali^19,20^ confirmed that layered composites are superior to conventional blended ones. Sonsilphong et al.^7^ reported that a W/Bi layered sheet with a thickness of only 0.14 mm (two layers, W/Bi thickness ratio of 0.8) had the same shielding efficiency as a standard lead plate (0.5 mm), even though the density of the former was 36% lower. The shielding efficiency of W/Bi layered composites varies with the X-ray energy. The layer thickness ratio is directly dependent on the complementary effect of the absorption edges. This is with keeping with the results reported by Sazali et al., who found that the complementarity effect of the absorption edges matches the layer thickness ratio^20^. Although the two-layer and three-layer metal materials show good X-ray shielding ability have been reported, these materials still lack of good flexibility and comfortable wearability. Therefore, flexible, lightweight and wearable X-ray shielding composites are urgently needed.

In this study, in order to obtain high-efficiency, flexibility and lead-free X-ray shielding materials, the multilayered (W/ethylene-octene copolymer)/(Bi/ethylene-octene copolymer) composites were designed and fabricated. The shielding afforded by the multi-layered interfaces and the complementary effect of the absorption was also studied. The experimental results of this study should serve as guidelines for the fabrication of lead-free, lightweight, and high-efficiency wearable X-ray shielding materials.

## Experimental

### Materials

The POE sample (8150), which had an octane content of 25%, was obtained from Dow Chemical Co., Ltd. (USA). Its melt flow index and density are 0.8 g/10 min (215 °C, 2.16 kg) and 0.909 × 10^3^ kg/m^3^, respectively. Bismuth powder was purchased from Qinghe Aerospace Metal Material Co., Ltd. (China); its average particle size was 5 μm. Tungsten powder was purchased from Ningbo Nanomaterials Co., Ltd. (China); its average particle size was 1 μm.

### Sample preparation

The above-mentioned metal powders were selectively mixed with POE in a fixed ratio for 10 min in an internal mixer (Model 600p, Haake Rheomix) at 100 °C and 60 rpm. The mixture was then compressed into sheets at 120 °C using a hot-press machine (AutoFour/30H-12, Carver) for 10 min, and the sheet were then annealed at room temperature for 5 min. All the samples were shaped into thin sheets (10 × 10 cm^2^), whose thickness was 1 mm unless otherwise mentioned.

### Morphological and structural characterization

The fractured cross-sections of the samples were examined through field-emission scanning electron microscopy (FE-SEM; Hitachi High Technologies America, Inc.) in order to observe their layered structure and interfacial morphology and to determine the sizes of the filler particles.

### X-ray shielding test

An X-ray generator (Y.TU 450-D09, YXLON, Shanghai, China) was used for the tests. Low-energy photons were generated through the X-ray tube and subsequently made to pass through an Al filter (beam hardening) to increase their average energy. The applied current, voltage, and irradiation time were 10 mA, 150 keV, and 60 s, respectively, while the absorbed dose was fixed at 1 Gy (J/kg). The scattering of the X-rays was prevented by placing a lead plate beneath the specialty EBT film. With an increase in the energy of the incident photons, the change in the color of the EBT film became more pronounced. The degree of color change was quantified using a high-performance color scanner (Expression 10000XL, Epson). Based on the numerical values of various points in the scanned image, their colors could be converted into the corresponding optical densities. The optical density is a quantifiable parameter and hence has been for dosimetry.

### Underlying theory and computer simulations

X-rays will undergo absorption, reflection, and transmission when they interact with the target material. The refractive indices in the X-ray energy regime used for the calculations were obtained from the Physical Measurement Laboratory, NIST^21^. The absorbed dose (amount of energy absorbed per unit mass of the absorber medium because of radiation exposure) can be determined from the dose-optical density curves. Therefore, the shielding ability of polymer composites with respect to X-rays can be quantified using film dosimetry. Then, from the absorbed dose, the transmittance ratio (I/I_0_) can be calculated as follows:

1 $$I/{I_0} = \exp ( - \mu x)$$

2 $$\mu /\rho = \frac{1}{\rho x}\ln ({I_0}/I)$$

where I_0_, I, x, and μ are the initial dose (shielding material not used), penetrated dose, thickness of the shielding sheet, and linear attenuation coefficient, respectively. In addition to the transmittance ratio (I/I_0_), the linear attenuation coefficient, μ, (cm^−1^) is another index for evaluating the shielding ability of composites and is primarily used to elucidate the effects of the material thickness on the shielding ability. Simultaneously, the mass attenuation coefficient (μ/ρ, cm^2^/g) is also used to evaluate the shielding ability of materials. Its advantage is that the effects of the material density on the shielding ability can be separated from the other factors, and the contribution of the structural design on the shielding ability of the composite can be evaluated independently.

The software Geant4 was employed to simulate the photon transmission process based on the Monte Carlo model. For the simulations, the emission position of the photons was set to 1.1 cm along the z-axis of the material, while the x- and y-axis positions were selected randomly such that they lay within the range of the shielding material. Moreover, the emission direction was taken to be parallel to the z-axis. The simulations involved shielding 40 photons with random energies in the range of 0–100 keV.

## Results and discussion

### Shielding efficiency of polymer composites with W and Bi microparticles

**Figure 1:**
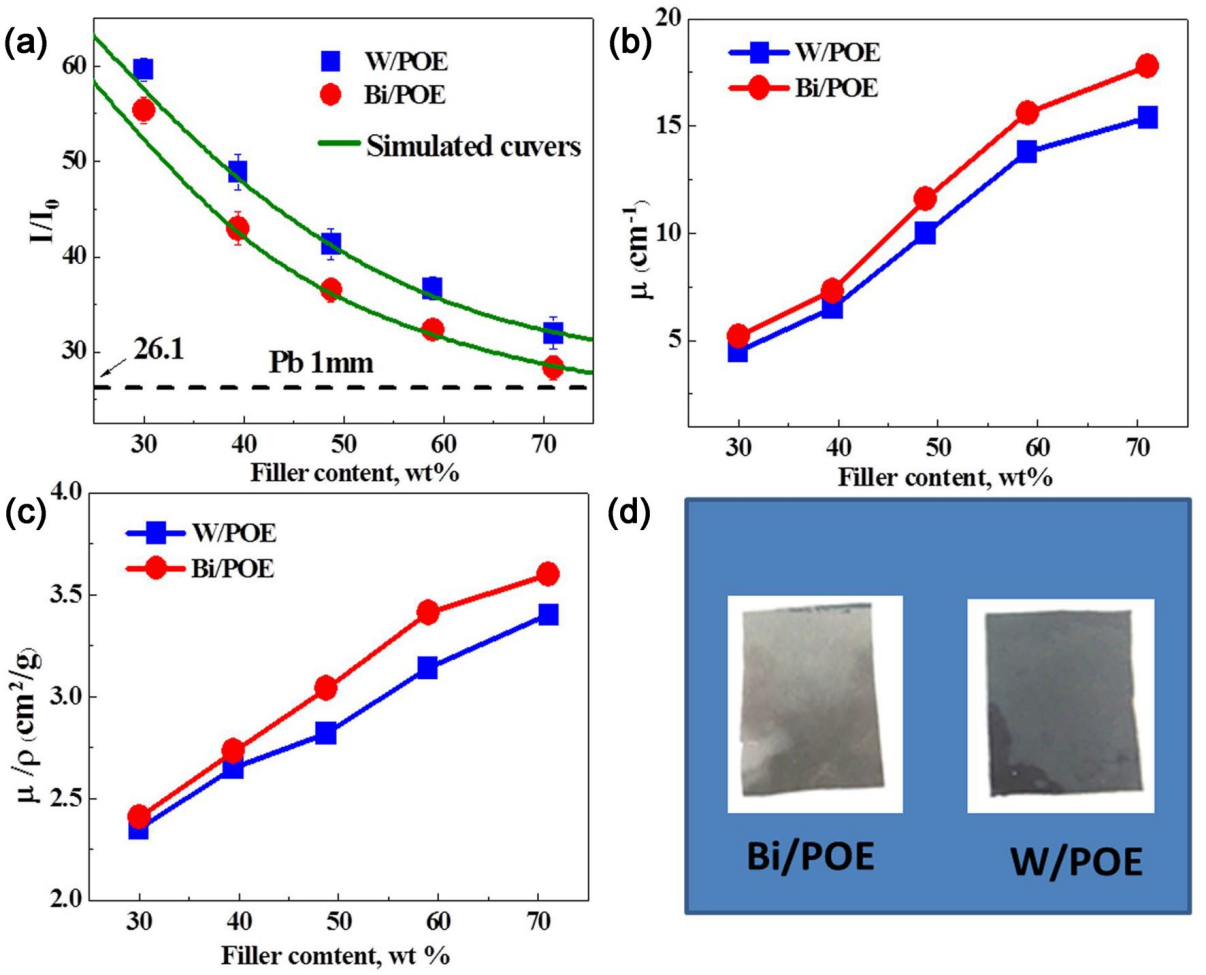
(**a**) Dose ratio (I/I_0_) of X-ray passed through the two composites (W/POE and Bi/POE). Correspondingly, the simulation curves were also displayed; (**b**) Linear attenuation coefficient versus the amount of metal content; (**c**) Mass attenuation coefficient versus the amount of metal content; (**d**) Digital images of the W/POE and Bi/POE composites.

The dependence of the transmittance of these composites on their metal filler content is shown in Fig. 1; the X-photon energy was 60 keV. With an increase in the metal filler content, the X-ray transmittance of the metal/polymer composites decreased sharply. When X-rays pass through a shielding material, they obey the following exponential attenuation law^21^:

3 $$I(x) = {I_0} \times B(x) \times \exp [ - \int_0^x {\mu (t)} d(t)]$$

where I (x) and I_0_ represent the intensity of the X-rays before and after they have passed through the shielding material, respectively; x is the thickness of the shielding material; μ is the linear X-ray attenuation coefficient of the material; and B(x) is the correction factor. It was assumed that the filler particles were homogeneously dispersed within the polymer matrix, and they were regular and spherical. The results of the simulations performed using Eq. (3) confirmed that the measured values were in keeping with the equation. Meanwhile, the Bi powder/polymer composites exhibited lower transmittances for the same filler content (sample thickness was 1 mm, and photon energy of X-rays was 60 keV). The linear attenuation coefficient and mass attenuation coefficient, both of which are significantly enhanced with an increase in the metal filler content, showed similar trends. These results were reflective of the intrinsic metallic characteristics of the W and Bi microparticles.

### Structural design of polymer composites

**Figure 2:**
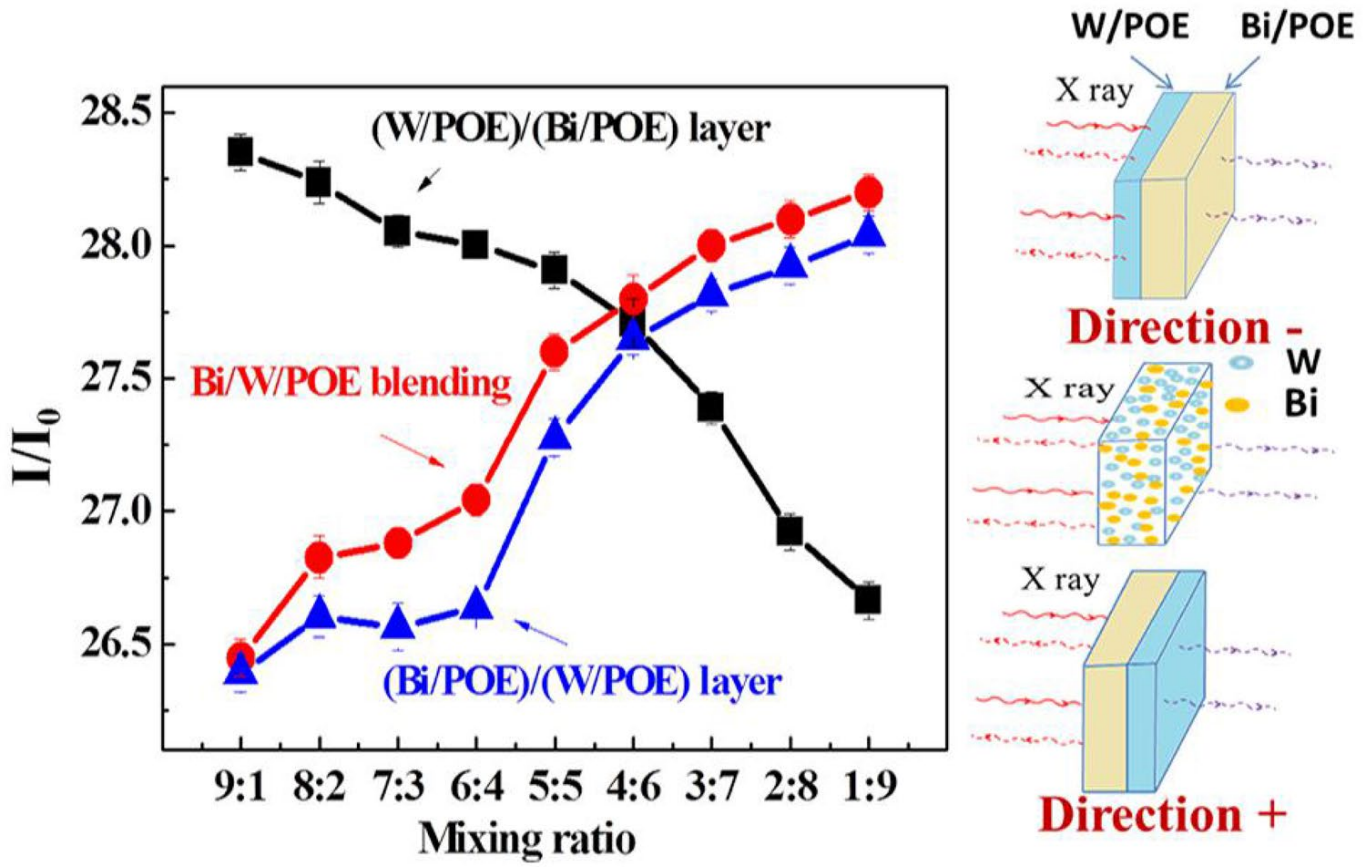
Dose ratio (I/I_0_) of X-ray passed through the blending and layered composites, and its sketch was also shown on the right side to clearly indicate the incident direction of X ray and structural differences of layered composites with different layer thickness ratio. When the X-ray firstly interacted with layered tungsten/polymer composites, this direction was defined as “Direction−”. Conversely, this direction was defined as “Direction + ”.

Layered polymer composites with different layer thickness ratios were prepared by hot-compression processing. Because of the layered-stack-like structure of the composites, the incident X-rays exhibited a high degree of directivity. The incident X-ray underwent two distinct types of interactions with the composites, depending on the direction of incidence: in the first case, the X-rays first interacted with the layered W/polymer part of the composites; this direction of incidence is called “Direction−”. In the second case, this direction is called “Direction+”. In order to elucidate the effects of the direction of incidence on the shielding efficiency, the X-ray shielding efficiency of blend composites composed of the same functional fillers and polymer was also measured and compared with that of the layered composites. As can be seen from Fig. 2, it was found that with an increase in the Bi content, the photon transmittance decreased sharply. In other words, the shielding efficiency was significantly enhanced. The atomic number of Bi is higher than that of W. Further, the Bi atom is larger than the W atom and hence is more conducive for inducing the photoelectric effect and dissipating the energy of photons. As a result, Bi-based composites exhibit excellent shielding ability for X-rays.

**Figure 3:**
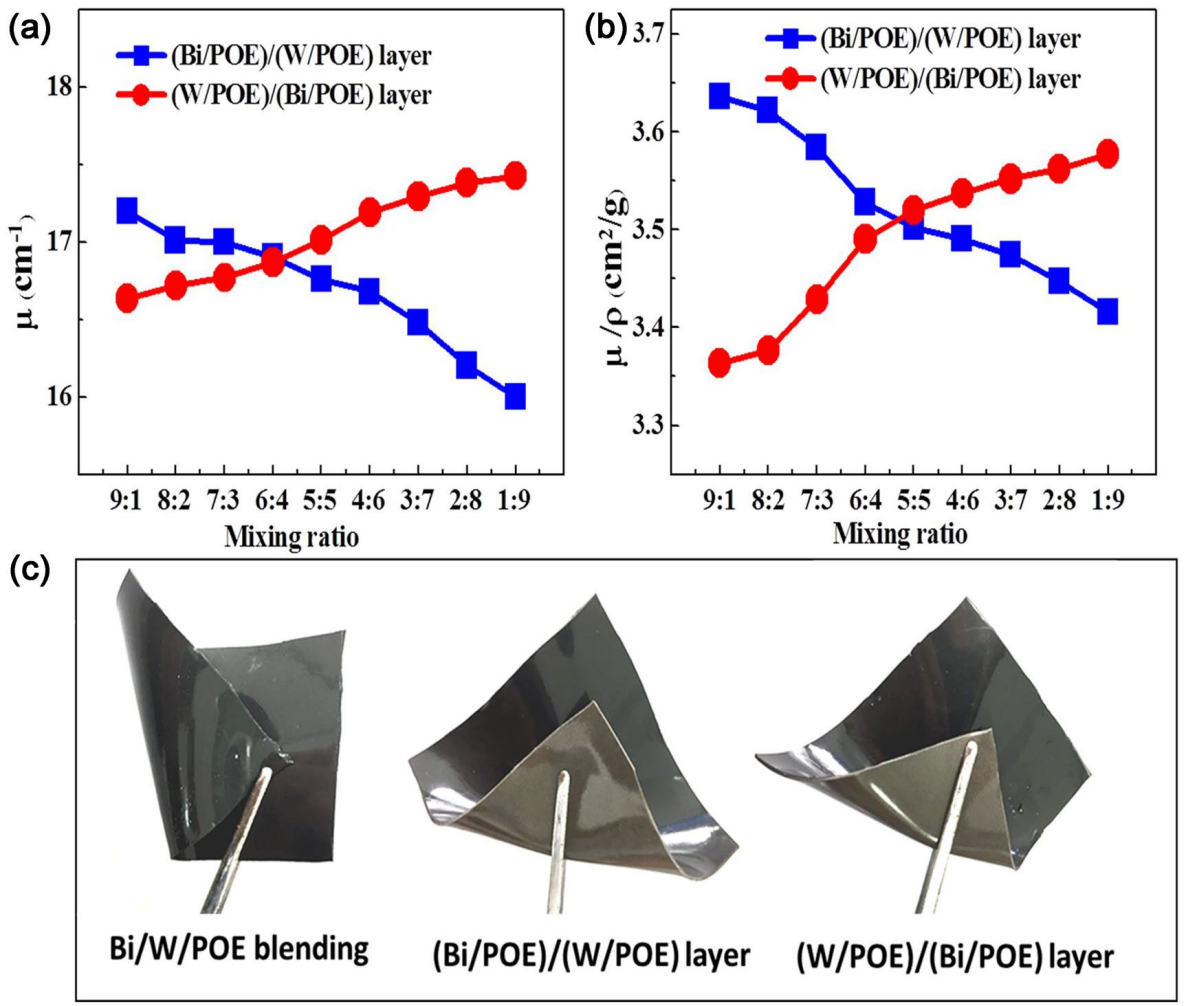
(**a**) Linear attenuation coefficient versus the layer thickness ratio; (**b**) Mass attenuation coefficient versus the layer thickness ratio; (**c**) Digital images of the flexible composites.

**Figure 4:**
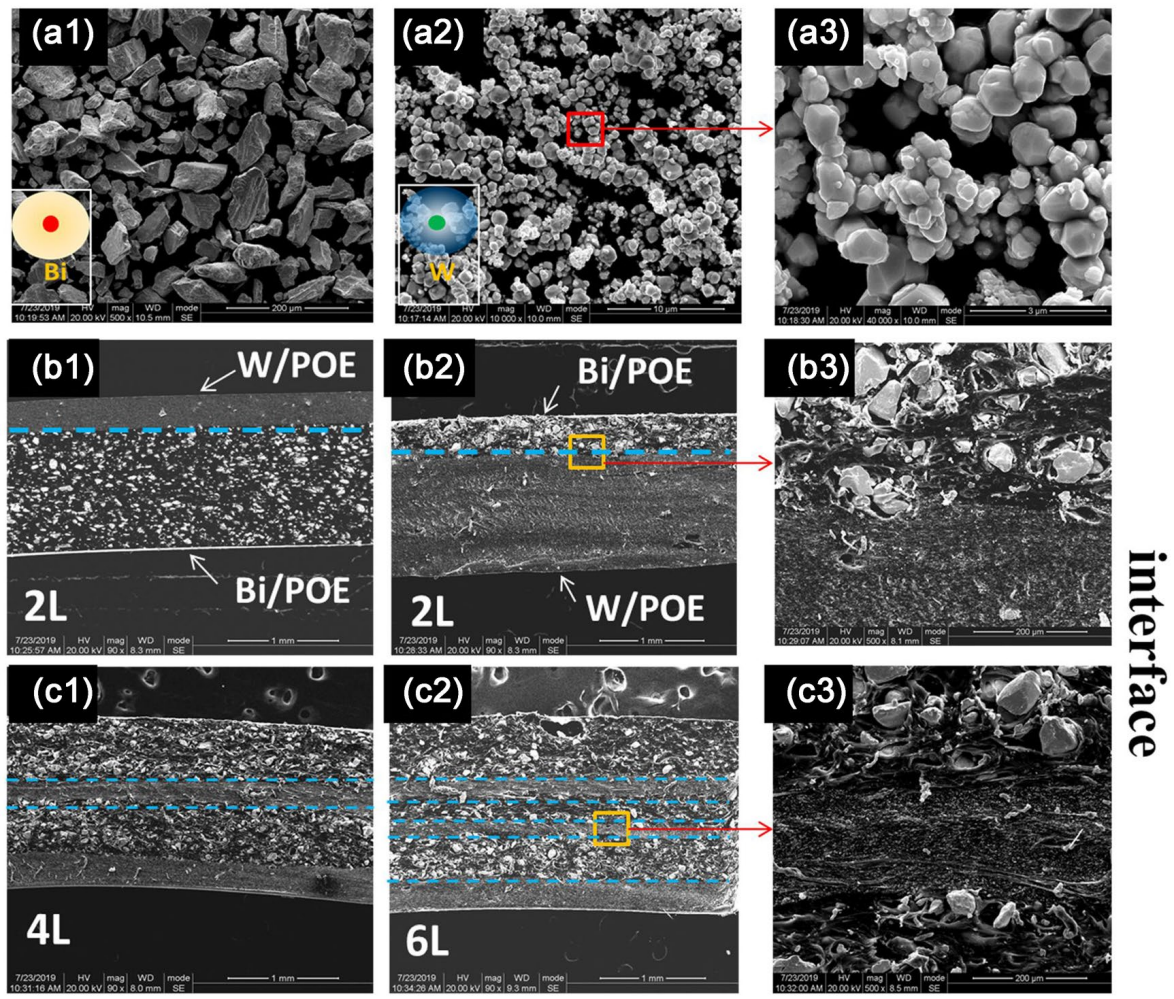
Scanning electron microscope (SEM) of bismuth (**a1**) and tungsten (**a2**) powders, and the local magnification image of the tungsten powders (**a3**); the (Bi/POE)/ (W/POE) layered composites (**b1**), and the layer thickness ratio was 7:3; the (W/POE)/(Bi/POE)layered composites (**b2**), and the layer thickness ratio also was 7:3; the cross section of alternating layered composites with 4 and 6 layers were shown in Figure (**c1**) and (**c2**);moreover, a local enlargement of the layered interface was shown in Figure (**b3**) and (**c3**).

Figure 3a,b shows the linear attenuation coefficient and mass attenuation coefficient as functions of the layer thickness ratio. Figure 3c shows digital images of the flexible composites. The photon transmittance of the blended composite when irradiated with X-rays was markedly higher than those of the layered composites, confirming that the layered structure aids photon attenuation. Simultaneously, the shielding efficiency along the X-ray incidence direction can be increased readily if the Bi particles (high Z number) are arranged in front of the W particles. The size distributions of the Bi and W particles as well as the layered composites with the different layer thickness ratios and the layered interfaces are shown in Fig. 4. It was found that the functional particles with a higher absorption edge should be arranged in front, so that the complementary effect of the different absorption edges can be exploited fully.

**Figure 5:**
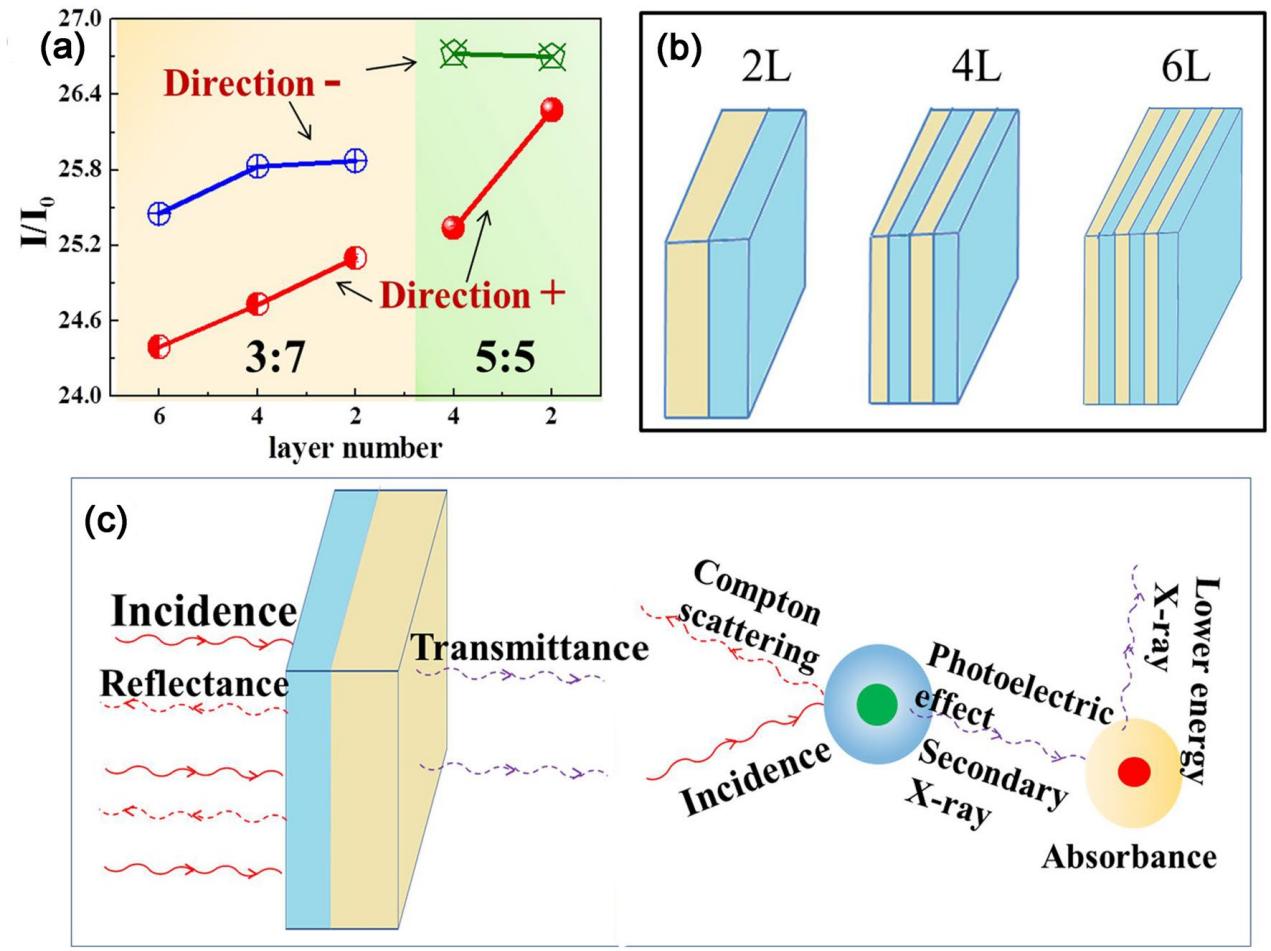
(**a**) The dependence of the X-ray transmittance on layer thickness ratios (4:6 and 3:7), incident direction and number layers. (**b**) the structural schematic of multilayered composite with different number of layers; (**c**) schematic diagram of photon attenuation mechanism.

A schematic of the multilayered composites with different numbers of layers and a schematic of the photon attenuation mechanism are shown in Fig. 5. When the number of layers of the multilayered composites is increased from 2 to 6 as shown in Fig. 5a, their X-ray transmittances decrease sharply. The higher the number of layers was, the greater was the decrease in the transmittance. Similarly, the degree of photon attenuation also increased when the high-atomic-number (high-Z-number) metallic particles were arranged in front in the multilayered composites, that is, in a manner such that the X-rays were initially incident on them. The multilayered structure increased the probability of the photoelectric effect occurring in several places within the composites. At the same time, it also caused the X-rays to be reflected and absorbed multiple times between the layers, resulting in further dissipation of the photon energy.

When the X-rays interact with the atoms of the shielding material, they undergo reflection because of Compton scattering, while secondary X-rays are created by the photoelectric effect (their energy is lower than the initial X-ray energy), as shown in Fig. 5c. On the one hand, the thickness of the shielding material can be increased in order to shield these secondary X-rays as well. However, this would also increase the weight of the shielding material. Hence, the best way of resolving this issue is to use a material with the appropriate absorption limit so that it can strongly absorb the radiation and effectively dissipate the energy of the incident photons. In this manner, the weight of the shielding material can be kept low, since, in this case, the shielding performance primarily depends on the complementarity of the absorption limits of the components of the shielding material and the probability of the photoelectric effect occurring. In this study, the multilayered structure of the fabricated composites ensured that the photoelectric effect occurred multiple times within the composites, thus greatly enhancing the complementary effect of the absorption limits. At the same time, the multiple interfaces within the multilayered structure ensured that the incident rays entered a two-dimensional layered space. Given the limitations of the multilayered space, the incident photons could not escape readily and underwent attenuation. Therefore, the X-ray shielding ability of the multilayer composites was higher because of the synergy between the two effects described above.

### Results of computer simulations

**Figure 6:**
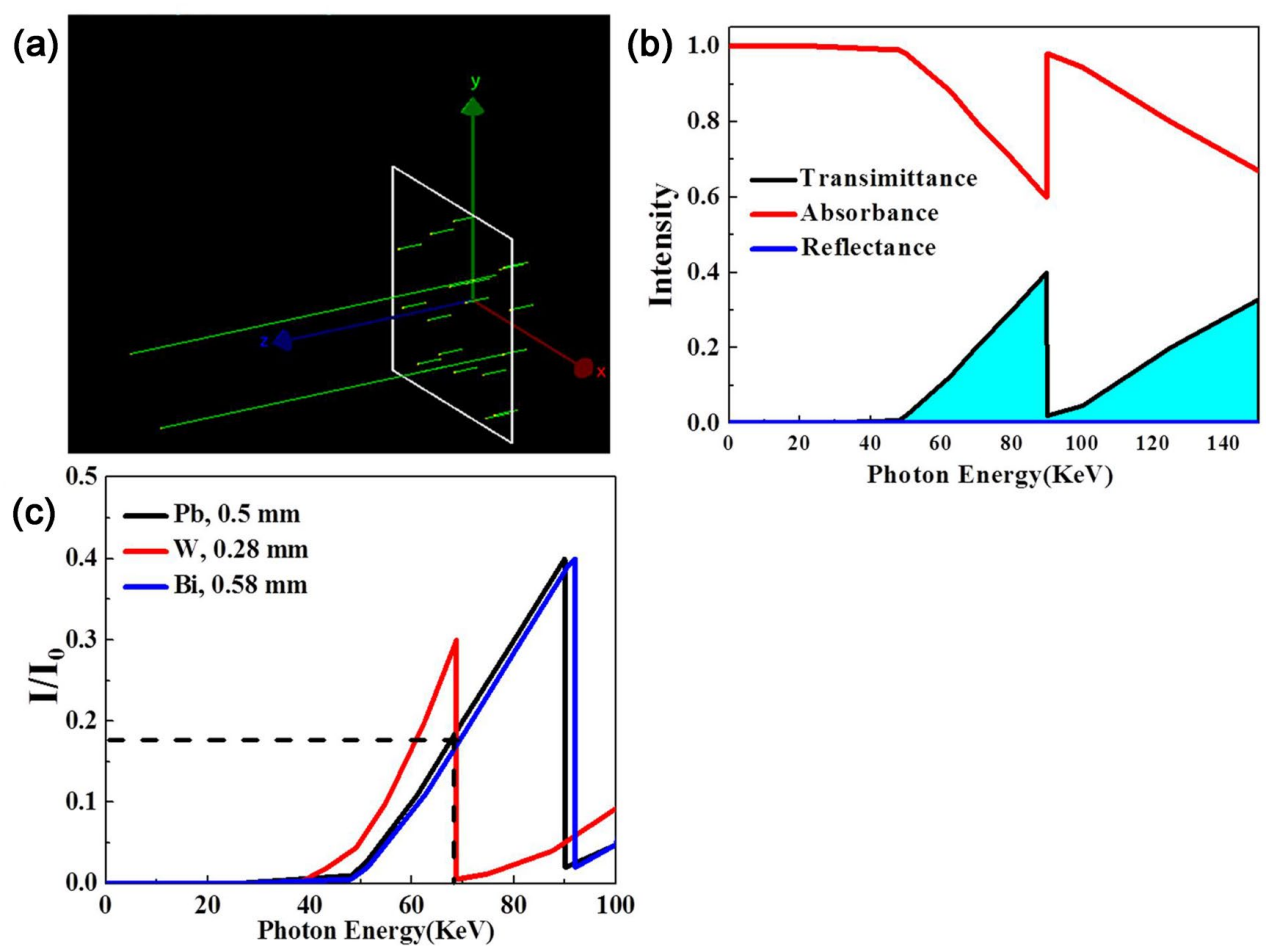
(**a**) The simulation process of shielding 40 photons with random energy in the range of 0–100 keV through shielding material; (**b**) Simulation results of reflection, absorption and transmission of lead materials. (**c**) X-ray transmission simulation results of lead, tungsten and bismuth pure metal materials.

It was difficult to fully analyze the passage of photons with different energy levels through the shielding material, given the limitations of practical testing methods. Thus, computer simulations were used to investigate the passage of photons with different energy levels through the polymer composites as shown in Fig. 6a. First, the X-ray shielding performance of a standard lead shield (thickness of 0.5 mm) was simulated, as shown in Fig. 6b. Since X-ray photons are low- and moderate-energy photons, their absorption is primarily dependent on the probability of the photoelectric effect occurring within the shielding material. Interestingly, the strong absorption of X-ray photons by lead at 90 keV results in a sharp increment in its shielding efficiency. This energy is the absorption edge or limit of lead. Simultaneously, the absorption blind zone of lead is 70–90 keV. Based on the Monte Carlo simulations performed in this study, we could determine the corresponding thicknesses of the investigated materials such that they would exhibit shielding efficiencies similar to that of a standard lead shield (thickness of 0.5 mm). The thicknesses for W and Bi were determined to be 0.58 and 0.28 mm, respectively, as shown in Fig. 6c. Similarly, W and Bi also showed different absorption edges at different energy levels. The absorption edges of W and Bi are such they can be used instead of lead-based materials in the 70–90 keV range. Thus, by combining these two materials, it is possible to make up for the shortcomings of lead-based shielding materials.

**Figure 7:**
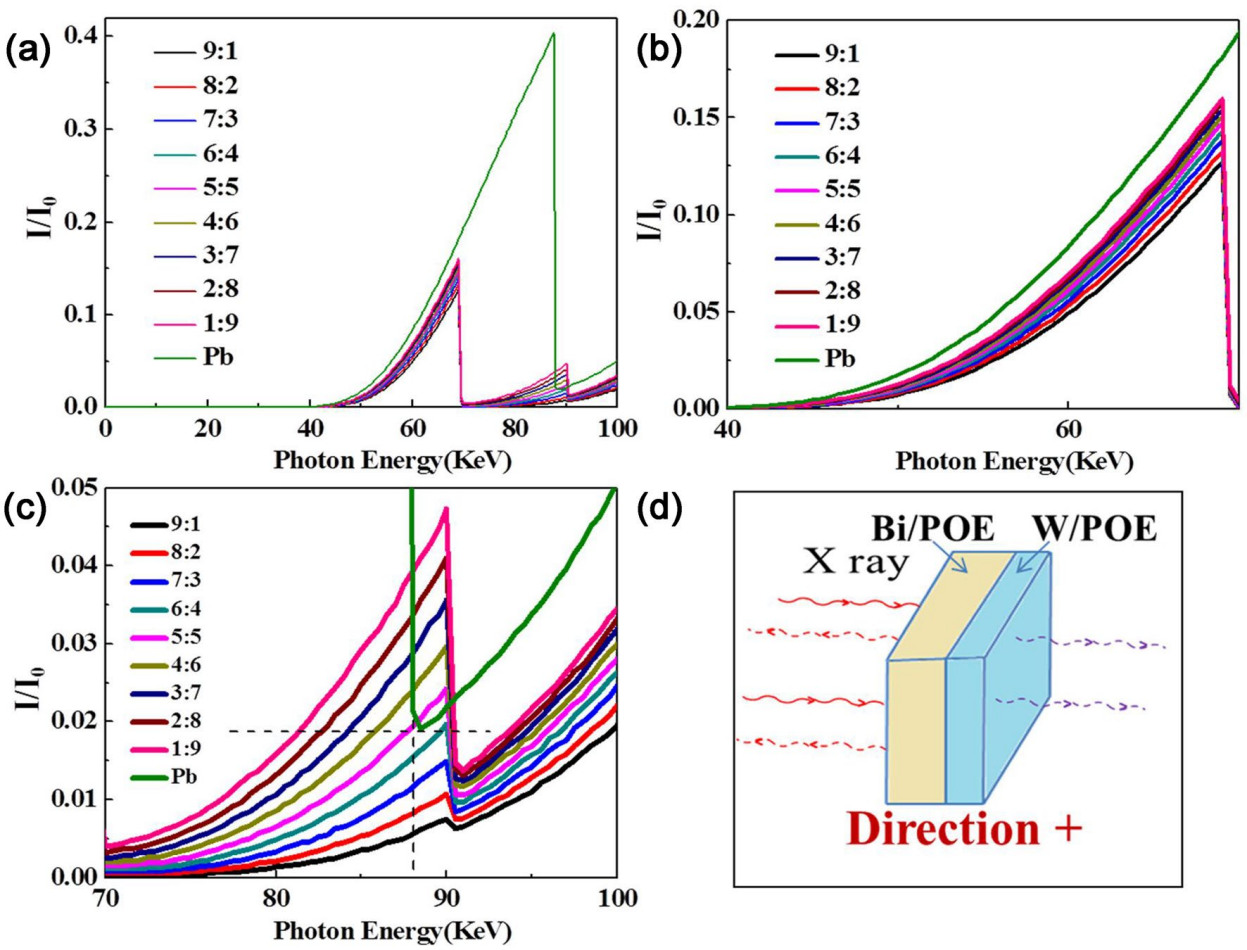
(**a**) X-ray transmission simulation results of the (Bi/POE)/(W/POE) layered composites in the range of 0–100 keV; (**b**) simulation results in the range of 40–70 keV; (**c**) simulation results in the range of 70–100 keV; (**d**) Structural sketch of the (Bi/POE)/(W/POE) layered composites, and incident direction was the “Direction + ”.

**Figure 8:**
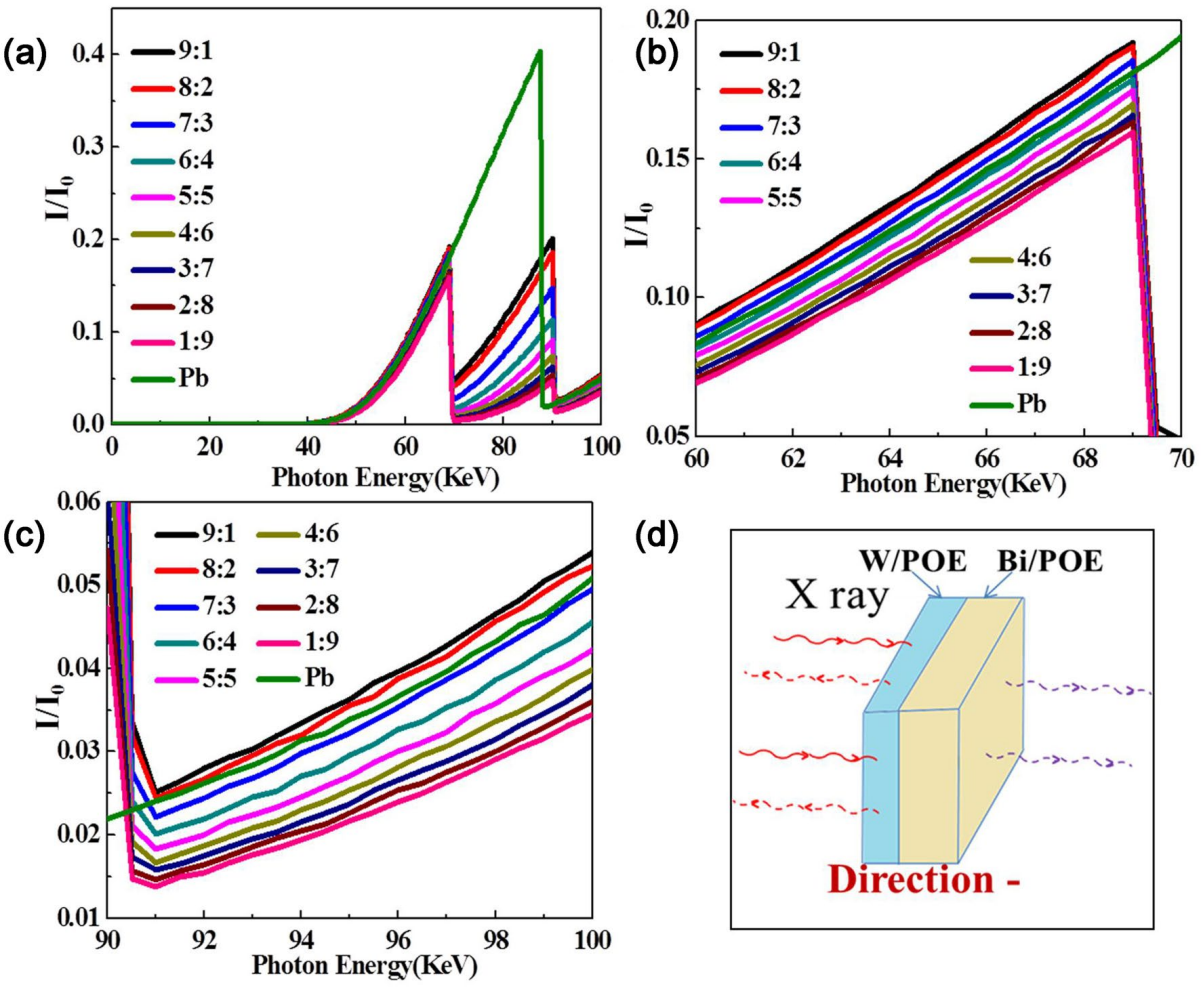
(**a**) X-ray transmission simulation results of the (W/POE)/ (Bi/POE) layered composites in the range of 0–100 keV; (**b**) simulation results in the range of 60–70 keV; (**c**) simulation results in the range of 90–100 keV; (**d**) Structural sketch of the (W/POE)/(Bi/POE) layered composites, and incident direction was the “Direction−”.

The X-ray shielding properties of the flexible and layered polymer composites with different Bi/W layer thickness ratios were simulated, and the results are shown in Figs. 7 and 8. Because the X-ray photons would have different directions of incidence, the interaction between the photons and the Bi/POE layer was simulated first. It was found that, in the energy range of 0–40 keV, the shielding efficiency was very high and almost the same irrespective of the mass ratios of W and Bi; Further, in the 40–70 keV range, the shielding efficiency of the layered composites was higher than that of lead. Finally, in the 70–100 keV range, when the layer ratio thickness of the Bi/POE layer to that of the W/POE was greater than 6/4, the shielding efficiency of the layered composites was significantly higher than that of lead. However, when the thickness ratio was lower than 6/4, the shielding efficiency of the layered composites was lower than that of lead for some energy levels (such as 88 keV). Because the W and Bi microparticles have different absorption edges at different energy levels, they could make up for the absorption blind zone of lead for energies of 70–90 keV. Therefore, by combining these two materials in layered composites, the disadvantages of lead-containing materials could be overcome. At the same time, the shielding efficiency was improved significantly.

The first interaction between the photons and the W/POE layer was also simulated, as shown in Fig. 8. It was found that, in the 0–40 keV range, regardless of the thickness ratio, the layered composites showed very high shielding efficiency. This result indicated that the layered composites could shield almost all the photons based on the photoelectric effect when the photon energy was less than 40 keV. In the 40–70 keV range, the shielding efficiency of the layered composites was better than that of lead. Finally, in the 70–88 keV range as well, the shielding efficiency of the layered composites was superior to that of lead and was enhanced significantly as the Bi content was increased. However, in the 88–90 keV range, the shielding efficiency of lead was better than that of layered composites regardless of the thickness ratio. Finally, in the 90–100 keV, when the thickness ratio was less than 7/3, the shielding efficiency of the layered composites was better than that of lead.

In the low-energy region, the penetration ability of the X-ray photons is low, and almost all the low-energy photons can be stopped. The directionality of the X-ray incident photons had an effect primarily in the 40–100 keV range. Hence, the observed differences in the shielding abilities can be ascribed to the absorption edges of the two metals used as the fillers in the composites. Therefore, the thickness ratio is a critical factor for ensuring that layered composites block photons even in the absorption blind zone of lead-containing materials and exhibit high X-ray shielding efficiency.

### Effect of number of layers on shielding efficiency

**Figure 9:**
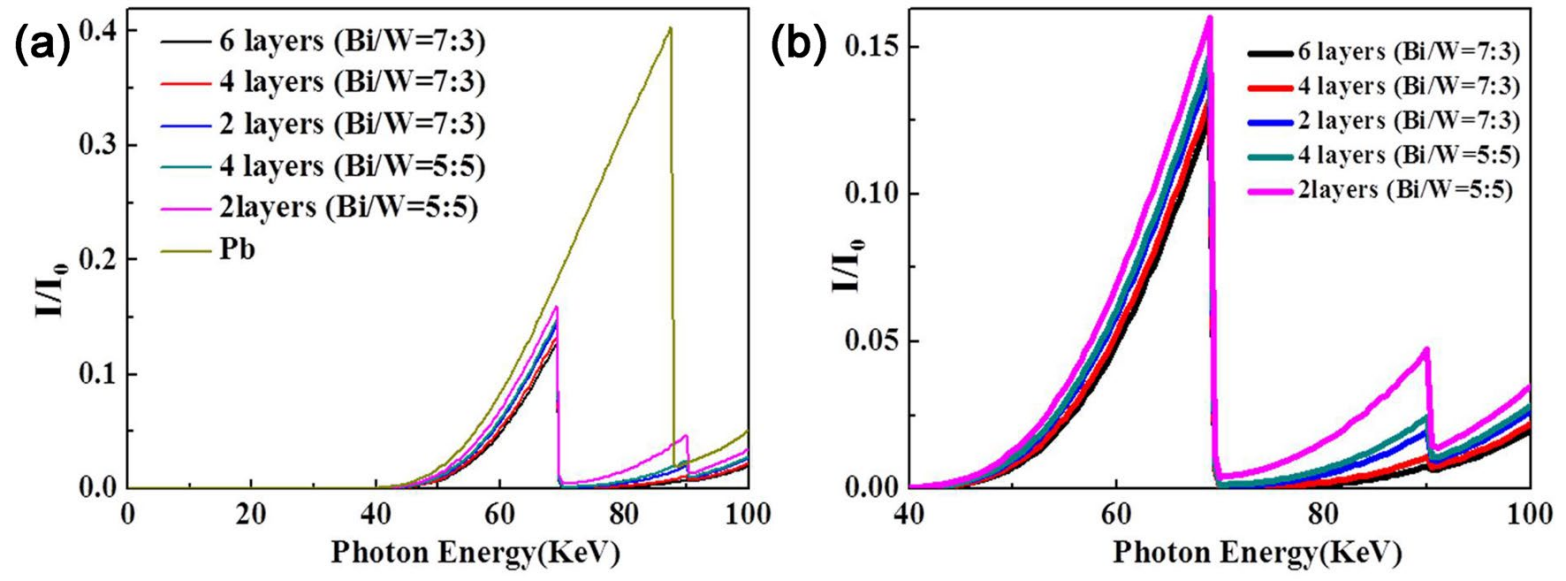
(**a**) The dependence of the X-ray transmittance on layer thickness ratios (7:3 and 5:5), incident direction and number layers through computer simulation; (**b**) the local magnification of (**a**) was presented in (**b**).

The shielding properties of multilayered composites with different numbers of layers in the case of photons with multiple energy levels were also simulated, as shown in Fig. 9. It was found that, in the 0–40 keV range, both lead and the layered composites exhibited very high shielding efficiency, as the energy of the incident photons was low. In the 40–70 keV range, the shielding efficiency of the layered composites was higher than that of lead. Moreover, it is worth noting that both an increase in the Bi content and an increase in the number of layers of the multilayered composites further reduced the photon transmittance. However, in the 88–90 keV range, the layered polymer composites with two layers and a layer thickness ratio of 5/5 exhibited the worst shielding capability. In the 90–100 keV range, the shielding efficiency of the layered composites was better than that of lead. The complete dissipation of the photon energy occurs because of the photoelectric effect. The constituent metals of the multilayered shielding composites contained a high number of free electrons and had multiple absorption edges, owing to which the photoelectric effect occurred multiple times within the materials, given their multilayered structure. This effectively enhanced the complementary effect of the absorption edges. At the same time, the presence of multiple interfaces within the multilayered composites also ensured that the incident rays entered a two-dimensional layered space. Given the limitation of space within the multilayered composites, the photons could not escape readily. Hence, the X-ray shielding ability of the multilayer composites was significantly better than that of lead.

**Figure 10:**
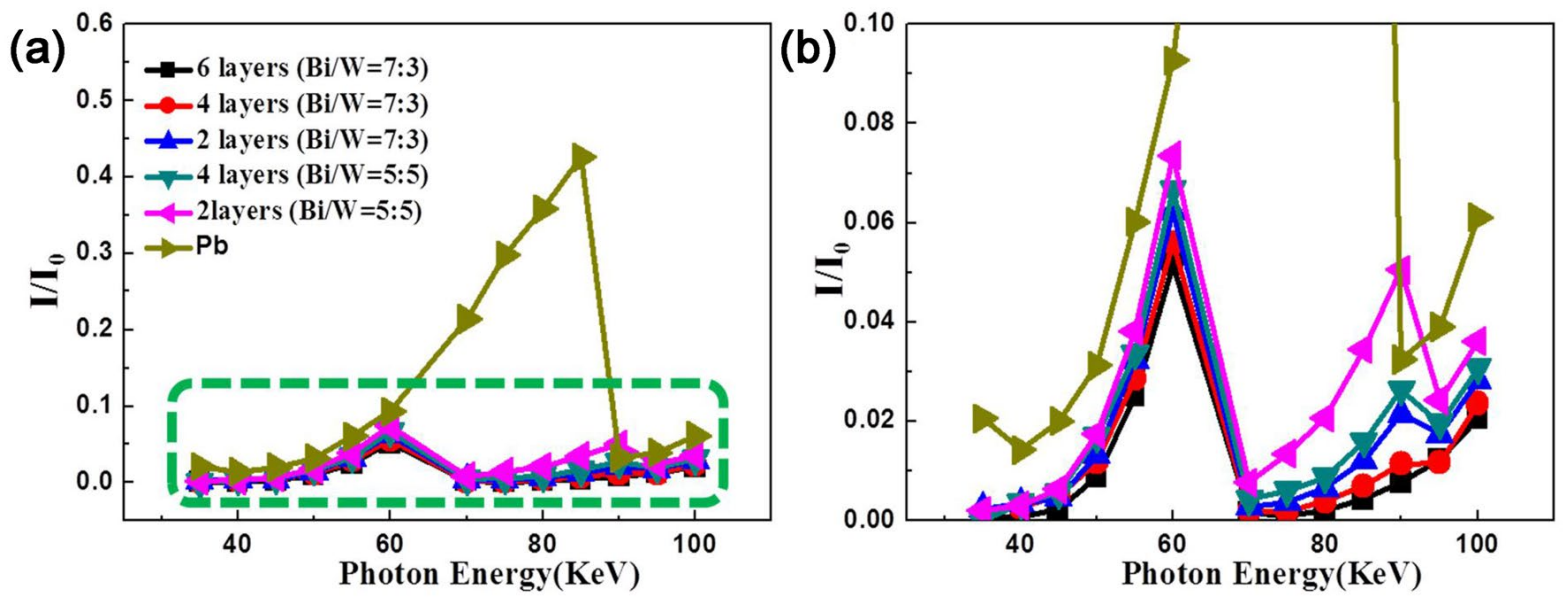
(**a**) The dependence of the X-ray transmittance on layer thickness ratios (7:3 and 5:5); the local magnification of (**a**) was also presented in (**b**).

In order to verify the simulation results described above, the X-ray shielding abilities of multilayer composites were tested for photons with different energy levels, as shown in Fig. 10. It was found that in the 25–45 keV range, the lead and layered composite samples all showed very high shielding efficiencies, as the photon energy was low. However, in the 45–90 keV range, the shielding efficiency of the layered composites was better than that of lead. The high Bi content and multilayered structure of the composites resulted in low photon transmittance and excellent X-ray shielding ability. However, in the 90–100 keV range, the shielding efficiency of lead was higher than that of the layered polymer composite with two layers and a thickness ratio of 5/5. Moreover, compare with traditional lead material and low-layer layered composites, the multilayered polymer composites (layer thickness ratio (POE/tungsten layer: POE/bismuth layer) = 3:7) with 6 layers had the lowest X-ray transmittance. In other words, the shielding ability of the multilayered polymer composites with 6 layers was superior over all these shielding materials. In order to show the X-ray shielding ability of alternating layered materials more clearly, the transmittance, linear attenuation coefficient and mass attenuation coefficient are summarized in Tables 1, 2 and 3, respectively. Interestingly, density of the multilayered polymer composites with 6 layers (7.81 g/cm^3^) was about 30% lighter than that of traditional lead materials (11.3 g/cm^3^).

**Table 1:** The dependence of the X-ray transmittance (I/I_0_) on layer thickness ratios (7:3 and 5:5) (1 mm thick).

Photon energy (KeV)	40	50	60	70	80	90
6 layers (Bi/W = 7:3)	0.00012	0.00719	0.04937	0.00041	0.00148	0.00749
4 layers (Bi/W = 7:3)	0.00015	0.00804	0.05297	0.00061	0.00214	0.01075
2 layers (Bi/W = 7:3)	0.00023	0.00977	0.05853	0.00119	0.0049	0.01965
4 layers (Bi/W = 5:5)	0.00025	0.01050	0.06095	0.00152	0.00661	0.02423
2 layers (Bi/W = 5:5)	0.00035	0.01286	0.06939	0.00411	0.01595	0.04727
Pb	0.00061	0.01750	0.07052	0.18493	0.31866	0.02729

**Table 2:** The linear attenuation coefficient (μ cm^−1^) of the X-ray transmittance on layer thickness ratios (7:3 and 5:5).

Photon energy (KeV)	40	50	60	70	80	90
6 layers (Bi/W = 7:3)	90.11	49.35	30.08	78.03	65.16	48.94
4 layers (Bi/W = 7:3)	88.12	48.23	29.38	74.09	61.47	45.33
2 layers (Bi/W = 7:3)	83.57	46.28	28.38	67.34	53.19	39.30
4 layers (Bi/W = 5:5)	82.98	45.56	27.98	64.89	50.19	37.20
2 layers (Bi/W = 5:5)	79.72	43.54	26.68	54.94	41.38	30.52

**Table 3:** The mass attenuation coefficient (μ/ρ cm^2^·g^−1^) of the X-ray transmittance on layer thickness ratios (7:3 and 5:5).

Photon energy(KeV)	40	50	60	70	80	90
6 layers (Bi/W = 7:3)	11.54	6.32	3.85	9.99	8.34	6.27
4 layers (Bi/W = 7:3)	11.28	6.18	3.76	9.49	7.87	5.80
2 layers (Bi/W = 7:3)	10.70	5.93	3.63	8.62	6.81	5.03
4 layers (Bi/W = 5:5)	10.90	5.99	3.68	8.41	6.60	4.89
2 layers (Bi/W = 5:5)	10.48	5.72	3.51	7.21	5.44	4.01

## Conclusions

The conclusions of the study can be summarized as follows: (1) By a multi-layered structural design, the absorption edges of the W and Bi microparticles could be synergistically made to overcome the absorption blind zone of lead in the 70–90 keV range. (2) The shielding ability of the fabricated multilayered composites could be effectively improved by exploiting the multiple complementarities of their absorption edges, which depended strongly on their layer thickness ratio. It was also found that the functional particles with a higher absorption edge should be arranged in front so that the complementarity of the absorption edges can be fully brought into play. (3) The multilayered structural design effectively weakened the probability of the X-ray penetration. Therefore, the X-ray shielding capability can be effectively enhanced through increasing number of layers and the synergistic effect of multi-layered interfaces.
